# Non-Pharmacological Interventions for Pain Management in Hemodialysis: A Narrative Review

**DOI:** 10.3390/jcm12165390

**Published:** 2023-08-19

**Authors:** Mustafa Ali Kassim Kassim, Alexandru Cosmin Pantazi, Wassan Nori, Liliana Ana Tuta, Adriana Luminita Balasa, Cristina Maria Mihai, Larisia Mihai, Corina Elena Frecus, Vasile Valeriu Lupu, Ancuta Lupu, Antonio Andrusca, Andra Maria Iorga, Radu Mihai Litrin, Irina Ion, Elena Ciciu, Sergiu Ioachim Chirila, Tatiana Chisnoiu

**Affiliations:** 1 Faculty of Medicine, “Ovidius” University of Constanta, 900470 Constanta, Romania; 2 College of Medicine, Mustansiriyah University, Baghdad 10052, Iraq; 3 Clinical Emergency Hospital of Constanta, 900591 Constanta, Romania; 4 Faculty of Medicine, “Grigore T. Popa” University of Medicine and Pharmacy, 700115 Iasi, Romania

**Keywords:** pain management, hemodialysis, acupuncture, cognitive behavioral therapy, relaxation techniques, virtual reality

## Abstract

This narrative review aims to summarize non-pharmacological interventions for pain management in hemodialysis patients, assessing their potential benefits and limitations in enhancing patient well-being and quality of life. We reviewed the current literature on five primary non-pharmacological interventions: acupuncture, cognitive behavioral therapy, relaxation techniques, virtual reality, and alternative methods such as transcutaneous electrical nerve stimulation, music therapy, and aromatherapy. We analyzed the evidence regarding their effectiveness, feasibility, and optimal implementation strategies. The existing evidence supports the potential benefits of these interventions in managing pain and improving the well-being of hemodialysis patients. However, further high-quality research is needed to confirm their effectiveness, establish implementation best practices, and assess their long-term impact on patient outcomes. Non-pharmacological interventions hold promise for pain management in hemodialysis patients. Additional research is required to optimize these interventions and validate their effectiveness, contributing to comprehensive pain management strategies for this vulnerable patient population.

## 1. Introduction

Hemodialysis is a life-saving treatment for people with end-stage renal disease (ESRD) that involves the removal of toxins and excess fluids from the blood using an artificial kidney. Although hemodialysis is essential to maintain the health of ESRD patients, the process can cause significant pain and discomfort, leading to a lower quality of life [1,2]. Pain faced by hemodialysis patients can be categorized into three categories: (a) procedure-related pain, (b) access-related pain, and (c) pain secondary to renal disease or its complications [3,4]. Effective pain management is crucial during hemodialysis to improve patient compliance, enhance the overall treatment experience, and promote better physical, mental, and emotional well-being [5,6].

Inappropriate pain management can lead to nonadherence to treatment, increased healthcare costs, and reduced quality of life [7]. Furthermore, unmanaged pain can exacerbate existing co-morbidities and contribute to the development of new health problems such as depression, anxiety, and sleep disturbances [8]. Managing pain in hemodialysis patients is often challenging due to the complex nature of ESRD and the potential risks associated with pharmacological interventions. Commonly prescribed medications for pain management include opioids, non-steroidal anti-inflammatory drugs (NSAIDs), and acetaminophen [9]. However, in 2016, the Centers for Disease Control and Prevention published guidelines that addressed the impact of opioids on patients suffering from chronic non-malignant pain, including ESRD. These guidelines recommended the reduction of dosages or discontinuation of opioid medications to alleviate the burden experienced by patients [10]. Moreover, those conventional medications do have other limitations, such as drug–drug interactions, the potential for addiction, gastrointestinal complications, and the need for dose adjustments due to decreased renal function [5]. Given that studies evaluating non-pharmacological approaches in patients with ESRD are scarce, in addition to the limitations of pharmacological approaches, this study was designed to review the available evidence on the effectiveness, feasibility, and accessibility of various non-pharmacological interventions (NPIs), including acupuncture, cognitive behavioral therapy, relaxation techniques, virtual reality, and other alternative methods in ESRD cases, and to evaluate their performance and application in clinical practice.

## 2. Methods

**Figure 1:**
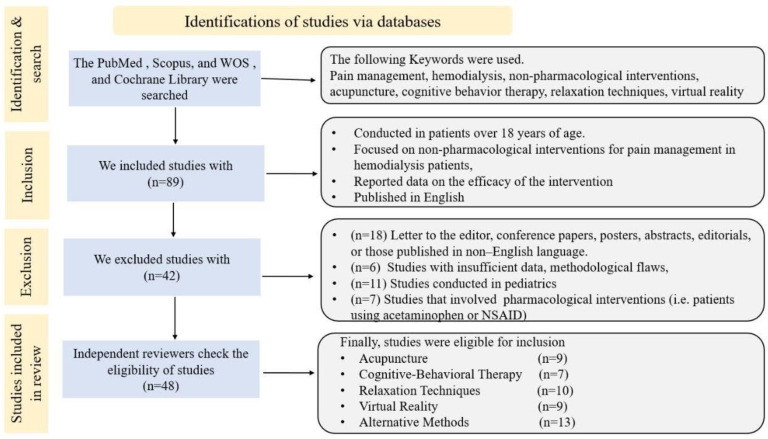
The study workflow.

This narrative review aims to synthesize the evidence on NPIs for pain management in hemodialysis patients. The methods followed a simplified approach to ensure a comprehensive analysis of the available literature, consisting of the following steps: a strategy to locate pertinent publications was performed through digital repositories, encompassing databases such as PubMed, Scopus, Web of Science, and the Cochrane Library. The search strategy used a combination of keywords and subject headings related to NPIs, pain management, and hemodialysis patients. Keywords were combined using Boolean operators such as “AND” and “OR” to refine the search results. The search was carried out for articles published up to 2023. The study workflow is described in Figure 1. In this review, we primarily focused on studies that reported reductions in pain and improvements in quality of life (QOL) among hemodialysis patients. We excluded any studies that examined the effects of pharmacological drugs, as they were beyond the scope of our analysis. For each study included, we collected information about the authors’ names, year of publication, study type, population, and sample size. This review aimed to summarize the principal mechanism of NPI action and its role in the following: 1. Improving patients’ QOL and related stress; 2. reducing the pain scale; and 3. the specific type of pain for which it was employed, whether this was procedure-related pain, access-related pain, or pain secondary to renal disease and its complications.

## 3. Acupuncture

**Figure 2:**
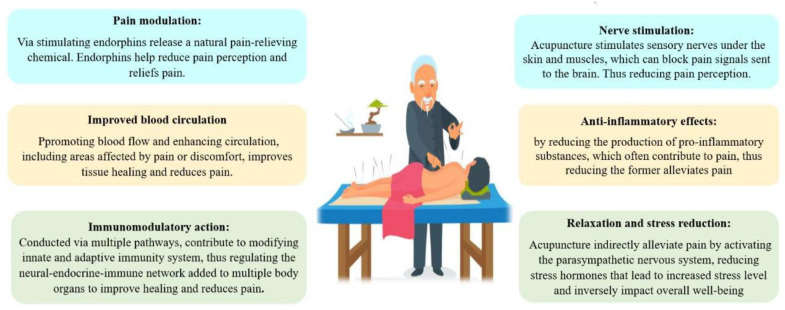
The main proposed pathways by which acupuncture interferes with pain intensity.

Acupuncture is a traditional Chinese medicine technique involving the insertion of thin needles into specific points on the body to stimulate the flow of energy (Qi) and restore balance [11,12,13]. The therapeutic effect is believed to be achieved through the modulation of multiple physiological systems and biochemical pathways, as shown in Figure 2, which describes how acupuncture contributes to pain reduction in ESRD [14,15,16].

Several studies have investigated the efficacy of acupuncture for pain management in the hemodialysis group. A recent randomized control trial by Correia et al. [17] in 2022 was conducted on 67 male and female adult patients who presented decreased functional capacity associated with hemodialysis. The study concluded that patients undergoing hemodialysis demonstrated improvements in functional capacity and muscle strength after acupuncture treatment. Similarly, another study was conducted by Bullen et al. [18] on 101 patients with end-stage renal disease, but this time acupuncture was associated with a massage procedure. The conclusion of this study was an improvement in the overall health-related quality of life of the 101 patients, which is confirmed by a new study that revealed that acupuncture was found to enhance the health-related quality of life in individuals undergoing maintenance hemodialysis following their treatment [19]. In addition, a study by Tsai et al. [20] focused on the impact of acupuncture on the reduction of restless leg syndrome (RLS) in hemodialysis patients.

RLS is a common complication that can negatively affect sleep quality and overall well-being. The study revealed that acupuncture treatment significantly decreased RLS symptoms but did not enhance sleep quality in patients on hemodialysis [20]. A systematic review and meta-analysis of seven randomized controlled trials conducted in 2023 showed that acupuncture is a safe and effective treatment for uremic pruritus (UP) in patients undergoing hemodialysis [21].

A randomized control trial conducted in 2020 on 50 patients divided into two groups of 25 each to determine how acupuncture can reduce pain and improve the quality of life in hemodialysis patients [22]. The study concluded that there was a great improvement in the groups who underwent the procedure and had a remarkable improvement in their overall quality of life. Furthermore, a pilot study investigated the feasibility, safety, and potential benefits of acupuncture for the management of patients who have hemodialysis sessions [23]. Twenty-four participants received individualized acupuncture treatments twice a week for six weeks, and their symptoms were assessed using the MYMOP2 Questionnaire and KDQOL-SF™ Version 1.3. The results showed a significant improvement in the most bothersome symptoms and some quality-of-life subscales at 7 and 11 weeks [23]. Acupuncture combined with hemodialysis was also more effective in relieving pruritus than hemodialysis alone [24]. Adverse events were rare, and acupuncture was found to be a safe treatment modality for patients with UP who received hemodialysis. Furthermore, in the context of fatigue experienced by hemodialysis patients, Melo et al. [25] revealed that acupuncture can offer a positive influence. Their literature review found a significant reduction in reported fatigue levels in patients treated with acupuncture, demonstrating the potential role of acupuncture in managing this debilitating symptom. Furthermore, they found that acupuncture can reduce sleep problems and HRQOL in patients with CKD [25].

However, while these studies collectively suggest that acupuncture can have multifaceted benefits for hemodialysis patients, it is crucial to note that acupuncture must be performed by qualified professionals to ensure patient safety. As reiterated by Xu et al. [26], acupuncture-related adverse events can occur, although some of these events might be minor and transient when the procedure is conducted by adequately trained professionals in proper conditions. Zheng et al. [27] explored the potential of acupuncture for improving cardiovascular function in hemodialysis patients. In this study, acupuncture was associated with improvements in mild hypertension, one of the most common co-morbidities in these patients [27]. The exact mechanism through which acupuncture impacts cardiovascular function is still under investigation, but the findings suggest a promising avenue for further research [28]. Acupuncture’s multifaceted benefits make it a suitable intervention for pain secondary to renal disease or its complications [21,23,24]. However, more extensive and well-designed studies are required to solidify these findings and determine the optimal treatment parameters for this patient population.

## 4. Cognitive Behavioral Therapy (CBT)

Cognitive behavioral therapy (CBT) is a goal-oriented psychotherapy that focuses on identifying and modifying maladaptive thoughts, emotions, and behaviors [29]. In the context of pain management, CBT aims to help patients develop effective coping strategies, improve pain tolerance, and improve self-efficacy [30]. CBT has been investigated for its potential to treat pain in hemodialysis patients. The systematic review and meta-analysis by Zegarow et al. [31] engaged in an exploration of cognitive behavioral therapy being employed as a psychological treatment strategy. It was reported that the lowering of the intensity of depressive symptoms in patients with hemodialysis. To improve patient well-being, they recommend considering incorporating cognitive behavior therapy as an extension to renal replacement therapy. This was in line with Zheng et al. [27] systematic review and meta-analysis that examined the benefits of CBT on depression and anxiety in hemodialysis patients. Furthermore, a randomized control trial conducted by Valsaraj et al. [32] on 67 chronic kidney diseases found that CBT is more effective than nondirective counseling in improving therapeutic adherence, as well as physiological and clinical parameters among CKD patients undergoing hemodialysis. At six months, the experimental group exhibited a significant reduction in interdialytic weight gain and blood pressure and an increase in adherence to dialysis, fluids, diet, and drugs [32].

A pilot study found that a tech-assisted cognitive behavioral therapy procedure for ESRD undergoing hemodialysis was feasible and well accepted [33]. While no significant changes in depression, fatigue, or pain were observed, preliminary results suggested that the intervention may improve physical health and pain. Additionally, a study explored the impact of a 12-week group CBT program, including mindfulness meditation, on the quality of life, mood, anxiety, perceived stress, and biochemical markers in seven ESRD patients undergoing hemodialysis and experiencing depression [34]. Measurements were taken at baseline and at weeks 8 and 12 using the WHOQOL-BREF, BDI-II, HAM-D, BAI, and PSS scales. Biochemical markers were measured at baseline and after 12 weeks. The results revealed significant improvements in quality of life, mood, anxiety, and perceived stress following the 12-week CBT program. In addition, serum creatinine levels significantly improved [34]. A randomized trial evaluated the impact of personal cognitive behavioral therapy in mitigating depressive symptoms and enhancing life quality for hemodialysis patients who exhibited pronounced depressive feelings [35]. A total of 59 patients from two New York dialysis centers completed the study and were assigned to a treatment-first group (*n* = 33) or a waitlist control group (*n* = 26). CBT was administered chairside during dialysis treatments for three months, with evaluations performed three and six months post-randomization. The treatment-first group experienced significantly greater reductions in depression scores (both self-reported and clinician-reported) compared to the waitlist group [35]. Furthermore, the treatment-first group showed greater improvements in quality of life and interdialytic weight gain. CBT has also shown positive results in improving the quality of sleep in patients undergoing hemodialysis [36]. CBT addresses the psychological aspects of pain [27,32].

In summary, CBT has shown potential for improving various aspects of well-being in hemodialysis patients, such as social support, quality of life, and mental health outcomes. Further research is needed, including exploring novel modalities such as Internet-based CBT, which could potentially enhance the accessibility and effectiveness of CBT interventions for this patient population. Another area to consider is establishing optimal implementation strategies and confirming CBT’s long-term benefits in hemodialysis patients.

## 5. Relaxation Techniques

**Figure 3:**
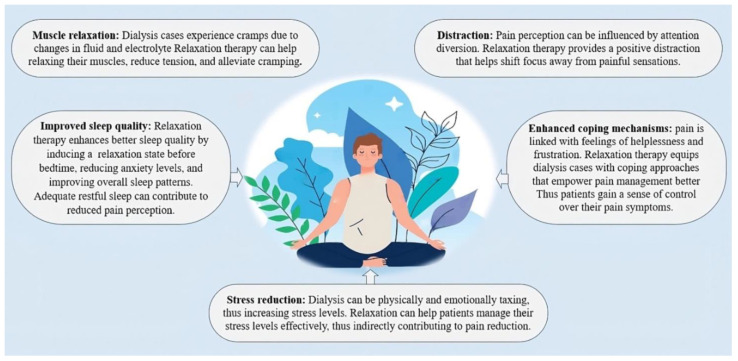
The main pathways by which relaxation techniques modify pain intensity.

Relaxation techniques encompass a variety of non-invasive approaches designed to promote mental and physical relaxation, thus alleviating stress and fostering well-being [37]. Examples of relaxation techniques include progressive muscle relaxation (PMR), guided imagery, deep breathing exercises, and mindfulness meditation [38]. These mechanisms for alleviating discomfort, as depicted in Figure 3, each follow their own unique pathways.

Numerous studies have investigated the potential benefits of employing relaxation techniques for pain management in individuals undergoing hemodialysis [39]. A quasi-experimental (time series) study evaluated the effects of increasing muscle relaxation on the intensity of restless leg syndrome (RLS) among adult patients on maintenance hemodialysis [40]. Sixty patients with RLS were divided into two groups, the study group receiving progressive muscle relaxation therapy. Results showed that the severity of RLS significantly decreased in the study group, with improvements observed in sleep patterns, physical activities, emotional well-being, and social activities [40]. A clinical trial, organized under randomized control, explored the impact of directed mental visualization on depressive and anxious symptoms, as well as vital signs, among individuals undergoing hemodialysis [41]. Eighty patients were randomly allocated either to a group undergoing guided intervention or a control group. Results displayed that the guided intervention group endured significantly reduced values of anxiety and depression after the intervention compared to the control group. In addition, there was a reduction in respiratory rate and heart rate in the intervention group [41]. A similar study aimed to assess the effect of muscle relaxation on foot pain in hemodialysis patients [42]. Ninety patients were randomly divided into control and experimental groups, with the experimental group practicing Benson muscle relaxation twice a day for a month. The results showed a significant decrease in pain intensity in the intervention group compared to the control, suggesting that muscle relaxation can effectively reduce pain in hemodialysis patients.

In 2023, a novel cluster-randomized controlled study was executed to gauge the efficacy of the Benson Relaxation technique in alleviating pain and perceived stress in patients undergoing hemodialysis [43]. The intervention group performed Benson’s relaxation twice a day for 10 min for 8 weeks, while the control group received an educational session about Progressive Relaxation. The study found that Benson’s Relaxation significantly relieved perceived stress and pain among hemodialysis patients after 1 week and 1 month of practicing, but not after only 1 week. The later study had similar results to a study conducted in 2014 that showed similar findings [44].

A study by Heidari et al. [45] conducted on eight patients and separated into two groups found that relaxation training using the Benson technique twice a day for four weeks was effective in lowering anxiety and pain experienced by hemodialysis patients. The intervention group showed significant improvements compared to the control group, and there was a correlation between pain perception and stress/anxiety levels. Moreover, a randomized controlled experimental investigation was undertaken with the intent to assess the impact of progressive relaxation exercises (PREs) on discomfort, exhaustion, and life quality among individuals receiving hemodialysis [46]. The study included 48 intervention and 48 control patients. The outcomes indicated that the average comprehensive fatigue score and the average pain score diminished in the group undergoing the intervention following the implementation of PREs. Meanwhile, the average ratings for both physical and mental elements of life quality showed an uptick (*p* < 0.05). No change was observed in the control group. The study reported that PREs can improve pain, fatigue, and quality of life in patients with hemodialysis [46].

Similarly, another study included 100 patients who received PMR every day for four weeks [47]. The results showed a decrease in pain level from moderate to mild, and a significant difference in pain level between the intervention and control groups (*p* < 0.001). The study showed that NPIs, such as PMR, can effectively reduce pain in hemodialysis patients. These techniques, by promoting relaxation and reducing anxiety, can be instrumental in alleviating procedure-related pain experienced during hemodialysis [42,43,47].

Relaxation techniques show promising potential in managing pain and improving the well-being of hemodialysis patients. However, more high-quality research is needed to confirm the effectiveness of these approaches and determine best practices for their implementation.

## 6. Virtual Reality

Virtual reality (VR) is an immersive technology that allows users to experience a computer-generated environment using head-mounted displays and other sensory devices [48]. In the context of pain management, VR has been used as a distraction technique to divert attention away from pain, reducing its perception and emotional impact [49]. While the evidence is still sparse, it holds encouraging potential for employing virtual reality in managing pain for individuals receiving hemodialysis [50,51]. A randomized controlled study evaluated the effects of combining exercise training with VR on functionality and quality of life in patients on hemodialysis [52]. The intervention group showed improved functional capacity and quality of life in physical and specific domains [52]. There was no significant impact on depressive symptoms. Furthermore, a pilot trial study tested the safety, acceptability, and utility of VR during hemodialysis treatment sessions using a new VR program that provided mindfulness training and guided meditation [53]. Patients on hemodialysis (*n* = 20) experienced the program on two separate occasions, and the results showed significant decreases in treatment and/or motion-related symptoms after VR exposure, with high levels of immersion in the VR environment reported. VR programs may be a safe platform to improve the experience of dialysis patients.

Another study conducted in Spain by Romero et al. [54] investigated the effects of virtual reality exercise on the functional and psychological states of patients having chronic kidney failure (CKF) undergoing hemodialysis treatment. The study randomized 80 patients into two groups: an experimental group that will use no immersive VR for intradialytic exercise and a control group that will exercise with a static pedal. The study concluded that virtual reality exercise can improve adherence to exercise in hemodialysis patients, leading to better outcomes such as improved inflammatory state, functional capacity, psychological state, and cardiovascular health. Additionally, VR can make hemodialysis sessions more bearable for patients.

A scoping review aimed to identify the potential of virtual reality interventions to affect the level of participation in self-care and quality of life in hemodialysis patients [55]. The review included 12 articles that demonstrated significant improvement in physical activity levels and a reduction in fatigue in patients during hemodialysis, with no adverse events. The study reported that virtual reality interventions may improve the level of adherence and engagement with treatment. Despite these promising results, more extensive research is needed to establish the effectiveness and feasibility of VR as a non-pharmacological intervention in this population [55]. Given its potential as a distraction technique, VR can be particularly beneficial in addressing procedure-related pain during hemodialysis sessions [54,55].

A focus should be made on evaluating the optimal frequency and duration of VR interventions, determining the most effective VR content for pain management in hemodialysis patients, and exploring the potential long-term benefits of VR therapy to patients’ overall quality of life and psychological well-being.

## 7. Other Alternative Methods

In addition to the previously mentioned NPIs, several alternative methods have been examined to manage pain in hemodialysis patients. These non-pharmacological approaches include transcutaneous electrical nerve stimulation (TENS), music therapy, and aromatherapy. TENS is a non-invasive method that involves delivering mild electrical currents through electrodes placed on the skin to help alleviate pain [56].

In a randomized controlled trial (RCT) conducted by Yang et al. [57], it was observed that TENS significantly reduced pain intensity in hemodialysis patients with access-related pain and increased salivary flow rate [57]. TENS can be a valuable tool in addressing access-related pain in hemodialysis patients. In various contexts, music was demonstrated to have a significant impact on pain reduction [58]. There are several hypotheses regarding the mechanism that underlies the musical effect. By engaging the brain in the processing of musical stimuli, the brain’s capacity to process pain signals may be diminished; this is called the distraction effect [59]. Listening to pleasurable and comforting music can encourage relaxation and positive emotions, which may relieve pain by reducing stress and anxiety; this is called emotional regulation [60]. Lastly, through neurochemical modulation, music induces the release of endorphins, which can act as analgesics to minimize pain sensations [61,62]. A study examined the effect of listening to patient-selected music on the pain and anxiety levels of hemodialysis patients during vascular access operations [63]. The experimental group listened to their favorite music during their operations, while the control group did not. The experimental group had drastically reduced subjective pain levels, objective pain behaviors, and lower levels of subjective anxiety and anxiety states compared to the controls [63]. These results suggest that music medicine can be a useful clinical intervention to reduce pain and anxiety in hemodialysis patients [63]. Similarly, another study used eight music therapy sessions using specific techniques, and patients were evaluated before and after the intervention [64]. They showed a significant reduction in depression symptoms and an improvement in quality-of-life dimensions such as functional capacity, pain, general health, vitality, mental health, list of symptoms and problems, and overall health [64]. The distraction effect of music can be especially useful in mitigating procedure-related pain during the hemodialysis process [63,64].

Aromatherapy is a type of alternative medicine that employs plant-derived essential oils to promote physical and mental health. While aromatherapy is not a pain remedy, it effectively reduces pain levels and enhances overall comfort [65]. Aromatherapy’s pain-relieving mechanism remains unclear, but multiple hypotheses explain its effectiveness. Aromatherapy activates the limbic system in the brain, which is connected to the olfactory system [66]. This stimulation causes the release of endorphins, a natural painkiller [67]. The delightful aroma of essential oils may distract the mind from painful sensations by acting as a diversion. Lastly, some essential oils possess anti-inflammatory effects, thereby reducing inflammation and relieving pain in afflicted areas [68]. Furthermore, a quasi-experimental study investigated the influences of sweet orange aromatherapy on pain and anxiety in 50 hemodialysis patients [69]. The results showed that patients who received sweet orange aromatherapy had significantly lower pain and anxiety scores than those who received calm breathing. Aromatherapy has shown promise in reducing access-related pain, such as during needle insertions [65,66,69].

## 8. Discussion

The current study embarked on a quest to investigate numerous non-pharmacological interventions for alleviating pain in individuals undergoing hemodialysis. Five principal strategies were identified. Among these, acupuncture demonstrated considerable promise as a benign and efficacious supplementary therapy for managing pain, enhancing functional capacity and muscular strength, lessening symptoms of restless leg syndrome and uremic pruritus, and bettering the QOL of patients on hemodialysis [13,19]. Nonetheless, additional investigations are imperative to confirm these results and define the ideal parameters for treatment. Cognitive behavioral therapy also exhibited the potential to ameliorate diverse facets of wellness in hemodialysis patients, including social support, QOL, and mental health outcomes [70]. CBT proved successful in attenuating depressive symptom severity, enhancing therapy adherence, and diminishing depression [31]. Internet-based CBT could potentially enhance the accessibility and effectiveness of these interventions for this patient population. Relaxation techniques, encompassing progressive muscle relaxation, guided imagery, deep breathing exercises, and mindfulness meditation, appear to hold potential in managing pain, fatigue, stress, and sleep patterns, as well as in promoting well-being and QOL for hemodialysis patients [43]. Further high-quality studies are needed to substantiate the effectiveness of these strategies and establish optimal procedures for their application.

Emerging as an innovative immersive technology, virtual reality has demonstrated potential benefits in pain management for individuals undergoing hemodialysis. This includes improved exercise adherence, enhanced functional capacity, improved cardiovascular health and psychological state, and making hemodialysis sessions more tolerable [53,54]. VR interventions may also promote patient compliance and engagement in treatment without any reported adverse events. However, more comprehensive research is required to validate VR’s efficacy and practicality as a non-pharmacological intervention in this demographic. Other alternative methods have also been explored for pain management in hemodialysis patients. TENS may reduce pain intensity, and music therapy can potentially reduce pain and anxiety and improve quality of life [71]. Finally, aromatherapy may reduce pain and anxiety during needle insertion [72]. These non-pharmacological approaches have demonstrated potential benefits, although more research is needed to confirm their effectiveness and establish best practices for their implementation.

## 9. Timing of Interventions

The application of non-pharmacological interventions in the context of hemodialysis varies in terms of their timing. It is essential to understand when these interventions are typically administered to appreciate their efficacy and relevance to the hemodialysis process.

*Acupuncture:* This ancient Chinese therapeutic technique is often scheduled adjacent to the hemodialysis session. Patients usually undergo acupuncture either preceding, following their hemodialysis sessions, or during the sessions to harness its full therapeutic potential, aiding in pain management and enhancing overall well-being [18,25]. 

*Cognitive behavioral* therapy sessions are generally organized independently of the hemodialysis procedure. They can be conducted on days when patients are not undergoing dialysis or after a session to address any psychological distress. All the included studies in our narrative review were introduced during the hemodialysis sessions [31,32,33]. 

*Relaxation techniques:* Techniques such as progressive muscle relaxation, guided imagery, and deep breathing exercises can be integrated both during the hemodialysis to alleviate immediate discomfort and outside the session to promote holistic well-being [44,47].

*Virtual reality*: Employed predominantly as an immersive distraction method, virtual reality interventions are typically introduced during the hemodialysis session [52,53]. This helps divert the patient’s attention from the immediate discomfort and creates a calming environment.

Other alternative methods: The application timing for these methods can be similar. For instance, music therapy can be introduced during the hemodialysis session to provide a soothing ambiance; similarly, aromatherapy might be more effective when used during the session to induce a state of relaxation [63,69].

The studies that explored the benefits of different non-pharmacological interventions are summarized in Table 1.

## 10. Side Effects of Non-Pharmacological Interventions

Before implementing NPIs for pain management in hemodialysis, clinicians must evaluate each patient’s unique requirements and preferences. Monitoring for adverse side effects and promptly addressing them can contribute to the safety and efficacy of these interventions. We have summarized the more frequent side effects reported in the literature in Table 2.

## 11. Study Limitations, Future Perspectives, and Further Research

Non-pharmacological interventions (NPIs) have gained popularity as an alternative or supplement to pharmacological treatments in recent years. While various studies have been conducted to investigate their usefulness, there are still significant gaps and deficiencies requiring further study.

While this review endeavored to offer an exhaustive overview of NPIs in the context of hemodialysis patients, it does come with certain constraints. Notably, individuals undergoing peritoneal dialysis were not encompassed in our analysis, marking a significant limitation. Additionally, the distinction between inpatient and outpatient status of the patients was not considered. Recognizing that the requirements and treatment modalities for hospitalized patients can substantially diverge from those receiving outpatient care presents another limitation of our study.

Much of the research has focused on the efficacy of specific NPIs; however, comparative studies that directly compare different treatments are needed to find the most effective pain management technique. The majority of NPI research focuses on short-term results. Future studies should focus on the long-term impacts to establish their viability and capacity to give long-term pain relief. Understanding the underlying processes through which NPIs relieve pain in hemodialysis patients is critical. Research should be conducted to understand better the physiological and psychological mechanisms of how NIP works to increase their efficiency [83]. When using NPIs in pain treatment, it is critical to consider patient preferences to ensure that they are acceptable and realistic in real-world situations. Since hemodialysis patients suffer discomfort from various underlying diseases, such as neuropathy or musculoskeletal difficulties, tailoring therapies should be considered [84,85]. More research has to look at whether adapting non-pharmacological therapies to specific patient characteristics improves pain management results. Another aspect to consider in NPIs is cost-effectiveness analysis, as some interventions endure upfront expenditures like training employees or acquiring equipment. Cost-effectiveness assessments can assist in establishing if these therapies are economically feasible in the long run when compared to pharmaceutical treatments [86]. The potential effects of combining NPIs are also worth exploring; future research in that area is warranted.

Finally, once the usefulness of NPIs has been shown, projects should concentrate on creating ways to incorporate them into routine clinical practice. Identifying challenges and facilitators, as well as defining criteria or protocols, can assist assure widespread acceptance and sustainability.

There is a lack of studies addressing the efficacy of analgesic medicine or comparisons between non-pharmacological interventions and an analgesic regimen. The current evidence regarding the efficacy and safety of NPIs seems promising; they are recommended and should always be available to hemodialysis patients. However, owing to the complexity of pain and the associated medical co-morbidities that most hemodialysis patients have, there is an urge for a cautious approach to managing pain.

## 12. Conclusions

In conclusion, a comprehensive review of the diverse NPIs for hemodialysis patients was performed. Preliminary data suggest the potential utility of these NPIs in mitigating pain and augmenting the QOL in this specific group of patients. However, a crucial need exists for high-quality research to validate their effectiveness, delineate optimal implementation methodologies, and investigate innovative approaches to enhance their reach and effectiveness.

It is essential to address the identified gaps in current knowledge. Progression in the domain of pain management for hemodialysis patients could potentially be driven by implementing comparative studies, analyzing long-term outcomes, and gaining insight into the precise mechanisms through which NPIs exert their effects. It is also important to consider patient preferences, evaluate cost-effectiveness, individualize interventions, and develop effective strategies for implementation. In the final analysis, a holistic, patient-centered approach to pain management, incorporating a variety of these NPIs, might be instrumental in addressing the unique needs of patients undergoing hemodialysis. This comprehensive strategy could lead to enhanced patient outcomes and improvement in the overall quality of healthcare provided to this specific patient group.

## Figures and Tables

**Table 1:** Comparative analysis on the benefits of different non-pharmacological interventions in hemodialysis patients.

Intervention	Authors; Year	Study Type	Participants	Perceived Benefits
Acupuncture	Kim et al. [16]; 2016	Observational pilot	24 hemodialysis patients	Reduction in pain related to hemodialysis
	Correia et al. [17]; 2022	Randomized control trial	67 male and female adult patients who are experiencing decreased functional capacity associated with hemodialysis	Improvements in functional capacity and muscle strength
	Bullen et al. [18]; 2018	Pre- and postintervention surveys	101 patients who have end-stage renal disease	Improvement in the overall health-related quality of life
	Correia et al. [19]; 2023	Randomized, controlled patient–assessor trial	67 cases receiving maintenance HD	Enhancement of the health-related quality of life in individuals
	Çevik et al. [22]; 2020	Randomized controlled trial	50 patients divided into two groups of 25, each consisting of patients on hemodialysis [1]	Improved overall quality of life. Acupressure significantly reduces extremity pain according to the VAS pain score
	Melo et al. [25]; 2020	Systematic review	Nine selected studies that examined patients with chronic kidney disease	Reduction in reported fatigue; reduced sleep problems and HRQOL in patients with CKD
Cognitive behavioral therapy (CBT)	Zegarow et al. [31]; 2020	Meta-analysis	226 patients with hemodialysis	Reduced intensity of depressive symptoms
	Valsaraj et al. [32]; 2021	Randomized controlled trial	67 patients with chronic kidney disease	Improved therapeutic adherence, as well as physiological and clinical parameters among CKD patients undergoing hemodialysis
	Jakbowski et al. [33]; 2020	Pilot study	10 ESRD undergoing hemodialysis was feasible and well accepted	No significant changes in depression, fatigue, or pain were observed
	Sohn et al. [34]; 2018	Pilot study	Seven ESRD patients undergoing hemodialysis and experiencing depression	Significant improvements in quality of life, mood, anxiety, and perceived stress following the 12-week CBT program. In addition, serum creatinine levels significantly improved
	Cukor et al. [35]; 2014	Randomized reciprocal trial	59 patients undergoing dialysis treatments for three months	Reductions in depression scores, greater improvements in quality of life and interdialytic weight gain
Relaxation techniques	Syam et al. [40]; 2022	A quasi-experimental trial	60 patients patients on maintenance hemodialysis	Reduced severity of restless leg syndrome and improvements observed in sleep patterns, physical activities, and social activities
	Beizaee et al. [41]; 2018	Clinical trial organized under randomized control	80 patients undergoing hemodialysis	Reduced values of anxiety and depression
	Blouchi et al. [42]; 2015	Random division into control and experimental groups	Ninety hemodialysis patients taking Benson muscle relaxation twice a day	A decrease in pain intensity in the intervention group compared to the control, suggesting that muscle relaxation can effectively reduce pain in hemodialysis patients
	Heidari et al. [45]; 2014	Randomized controlled trial	80 patients undergoing hemodialysis	Lowered anxiety and pain experienced in hemodialysis patients
	Kaplan et al. [46]; 2014	Randomized controlled experimental investigation	48 intervention and 48 control patients undergoing hemodialysis	Reduced fatigue score and average pain score and improved life quality
Virtual reality	Maynard et al. [52]; 2019	Randomized controlled trial	20 intervention and 20 control patients undergoing hemodialysis	Improved functional capacity and some quality-of-life domains of hemodialysis patients
	Hernandez et al. [53]; 2021	Pilot study	20 patients undergoing hemodialysis	Decrease in symptoms such as fatigue, nausea, lightheadedness, and headaches that often manifest during hemodialysis sessions
	Romero et al. [54]; 2023	Randomized controlled trial	40 intervention patients and 40 control patients	Improved adherence to exercise in hemodialysis patients leads to better outcomes, such as improved inflammatory state, functional capacity, psychological state, and cardiovascular health
Transcutaneous electrical nerve stimulation	Yang et al. [57]; 2019	Randomized controlled trial	80 patients undergoing hemodialysis	Reduced pain intensity in hemodialysis patients with access-related pain and improved salivary flow rate
Music	Kim et al. [63]; 2021	Randomized controlled trial	32 intervention patients and 33 control patients	Reduced pain and anxiety levels of hemodialysis patients during vascular access operations
	Hagemann et al. [64]; 2019	Interventional study	23 patients undergoing hemodialysis	Significant decrease in depression symptoms and improved quality of life across various dimensions, including functional capacity, pain, general health, vitality, mental health, and overall well-being
Aromatherapy	Reyes et al. [69]; 2020	A quasi-experimental	50 patients undergoing hemodialysis	Sweet orange aromatherapy was effective in reducing pain and anxiety in hemodialysis patients
	Yıldız et al. [72]; 2022	Systematic review	Seven studies included in the review	Decreased pain during the fistula needle intervention

**Table 2:** The side effects of non-pharmacological interventions.

No.	Non-Pharmacological Intervention	Reported Side Effects	Supporting Reference: 1st Author Name and Year
1	Acupuncture	Usually safe, but it can have mild side effects like bruises, bleeding, or pain where the needles were put in. Rarely, more major side effects like infections or organ damage can happen if you do not follow the right hygiene and approach.	Nielsen A et al. [73] in 2022 Dusek JA et al. [74] in 2022
2	Cognitive behavioral treatment	It is safe and well tolerated by most people and has few bad effects. Some people may feel temporary pain or mental distress during treatment meetings as they face and deal with problems linked to their pain.	Nees TA et al. [75] in 2020 Driscoll MA et al. [76] in 2021 Arcoraci V et al. [77] in 2021
3	Relaxation techniques	These are generally safe and have no major side effects. But, some people may find it hard to fully relax or get frustrated if they do not reach the level of relaxation they aim for.	Kesik G et al. [78] in 2023 Hargrove N et al. [79] in 2021 Ghanbari A et al. [80] in 2022
4	Virtual reality	It is usually safe to use, but patients with motion sickness may feel dizzy or sick because of the way the virtual world moves.	Martin JL et al. [81] in 2022 Hsieh, Chung et al. [82] in 2022

## Data Availability

No new data were generated.
